# Mediastinitis Secondary to Peripherally Inserted Central Catheter Migration and Perforation after Minor Trauma: A Case Report

**DOI:** 10.5811/cpcem.2020.11.49408

**Published:** 2021-01-27

**Authors:** Osvaldo Martinez, Justin Puller

**Affiliations:** University of Pittsburgh Medical Center, Hamot, Department of Emergency Medicine, Erie, Pennsylvania

**Keywords:** Peripherally inserted central catheter, catheter migration, catheter perforation, mediastinitis

## Abstract

**Introduction:**

The use of peripherally inserted central catheters (PICC) has been integral to the advancement of medical care in both in-patient and out-patient arenas.^1^ However, our knowledge of PICC line complications remains incomplete, particularly in regard to venous perforation and extraluminal migration. Utilization of displaced catheters harbors lethal complications and is an infrequently reported phenomenon, with traumatic etiologies only referenced as possible mechanisms; however, to date no formal cases have been reported.^5^,^6^

**Case Report:**

We report a case of a fall associated with extraluminal PICC migration and perforation causing mediastinitis and severe sepsis after total parenteral nutrition (TPN) infusion in a 54-year-old woman. Our patient required a right-sided PICC for long-term home TPN due to severe malnutrition following gastric bypass surgery. During a routine home care visit our patient was found tachypneic, hypoxic, and short of breath. Computed topography imaging in the emergency department (ED) identified the injury, likely related to the recent fall. The patient experienced a complicated hospital course after removal of the PICC. Although rare, PICC line migrations and perforations cause serious complications that should be considered by emergency physicians evaluating patients with chronic indwelling vascular access.

**Conclusion:**

Given the efficacy and widespread use of PICC lines, we present this case as a rarely reported but life-threatening complication that requires particular attention. Emergency physicians should be aware of such PICC line complications when encountering patients with chronic indwelling vascular access.

## INTRODUCTION

Peripherally inserted central catheters (PICC) have played an integral role to the advancement of medical care in both the in-patient and out-patient arenas.^1^ Prolonged therapies that traditionally required hospitalization, such as total parenteral nutrition (TPN), chemotherapy, and extended courses of antibiotics, can now be administered in outpatient facilities or in the home setting via PICC lines, providing convenience to patients and cost savings both to patients and hospitals.

Peripherally inserted central catheters are placed with ultrasound guidance by a dedicated intravascular team or under fluoroscopy by an interventional radiologist into the large vessels of the upper arm: the cephalic, basilic, or brachial veins. Optimal tip location is considered to be at the lower one-third of the superior vena cava or cavoatrial junction.

Peripherally inserted central catheters lines are generally well tolerated, although complications requiring prompt treatment can arise. Commonly cited frequencies of complications include deep vein thrombosis (30.6%),^2^ phlebitis (4–21%),^3^ catheter-related infections (3–5.7%),^3^ and late tip migration (1.5%).^3^ Tip migration, although uncommon, has been reported to occur within the first few days or months after insertion^3^ and is a major risk factor for PICC-related venous thrombosis.^4^

Venous perforation secondary to tip migration is a rare phenomenon that harbors potentially lethal complications. The frequency of PICC line perforations as a result of migration is not known, as few cases have been reported in the literature.^5^,^7^,^13^,^14^ Traumatic PICC line migrations and perforations are a particularly unusual entity, and to date there have been no reported cases. This report details a ground-level fall as the likely cause of PICC line migration and perforation, with resulting mediastinitis and severe sepsis.

## CASE REPORT

A 54-year-old woman with a right-sided PICC line placed in November 2015 presented to the emergency department (ED) two months later for evaluation of neck pain and hypoxia. She reported a history of Roux-en-Y gastric bypass in 2007 and revision in 2011 for recurrent marginal ulcers that required long-term TPN through a PICC secondary to severe caloric malnutrition. A visiting home health nurse found the patient short of breath, and hypoxic with oxygen saturations in the 70s percentile on room air. The patient’s daughter reported an unwitnessed, ground-level fall three days prior.

**Image 1:**
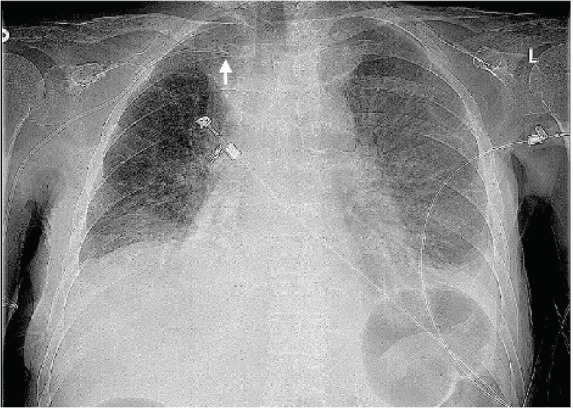
Radiograph identifying migrated, right-sided peripherally inserted central catheter with tip in proximal right subclavian vein (arrow) with bilateral pleural effusions.

Upon initial ED evaluation, patient vital signs were as follows: temperature of 36.4° Celsius, blood pressure of 79/54 millimeters of mercury, heart rate of 136 beats per minute, respiratory rate of 18 breaths per minute, and oxygen saturation of 89% on room air requiring two liters of supplemental oxygen via nasal cannula. Physical examination demonstrated a moderately sized, tender mass to the lower right anterolateral neck, just superior to the clavicle with mild overlying erythema and tenderness along the right trapezius muscle. Radiograph of her chest identified bilateral pleural effusions, with suboptimal location of the PICC line along the proximal right clavicle, several centimeters from the cavoatrial junction (Image 1).

**Image 2:**
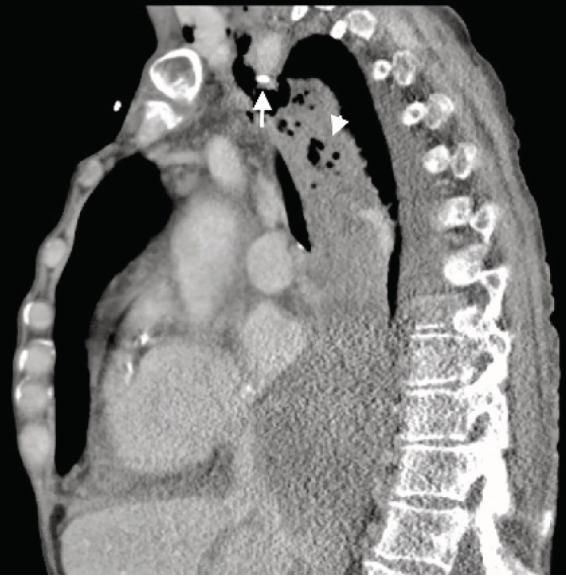
Sagittal computed tomography of the thorax demonstrating gas and fluid collections in posterior mediastinum (arrowhead) with peripherally inserted central catheter tip (arrow) terminating extraluminal in superior mediastinum.

Computed tomography (CT) of the thorax identified a significant amount of gas and fluid collections located in the soft tissues of the right side of the neck with extension into the mediastinum (Image 2).

**Image 3:**
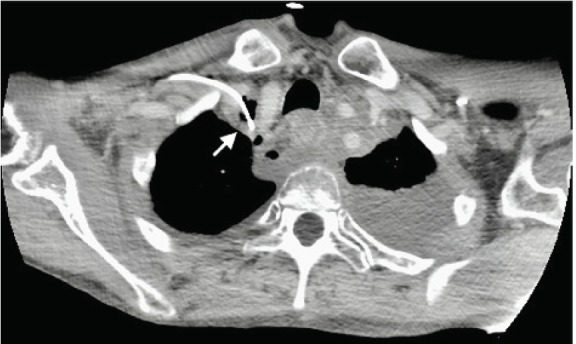
Transverse computed tomography thorax identifying peripherally inserted central catheter line perforation of right innominate vein, located superiorly and extraluminal in right upper mediastinum (arrow) with a large left pleural effusion.

Bilateral pleural effusions were re-demonstrated from chest radiograph, and the tip of the patient’s right PICC line was found to have perforated the right innominate vein, terminating extraluminal in the right upper mediastinum (Image 3). A non-occlusive thrombus was also found at the confluence of the right innominate vein.

**CPC-EM Capsule**

What do we already know about this clinical entity?

Out-patient use of peripherally inserted central catheters (PICC) is increasing due to convenience and safety profile but can harbor life-threatening complications.

What makes this presentation of disease reportable?

This is the first reported case of venous perforation due to traumatic migration of a PICC line in an out-patient setting causing severe morbidity.

What is the major learning point?

Emergency physicians should be familiar with both the common and rare highly morbid complications of PICC lines in patients with chronic indwelling vascular access.

How might this improve emergency medicine practice?

Differentials in patients with PICC lines should broaden to include mechanical and physiologic complications, with lower thresholds for advanced imaging and laboratory evaluation.

Laboratory work returned remarkable for evidence of severe sepsis with a leukopenia of 3.9 white blood cells × 10^9^/liters (L) (normal range: 4.5 – 11 × 10^9^/L) and a lactic acidosis of 4.1 millimoles (mmol)/L (0.5–1 mmol/L) requiring aggressive volume resuscitation and broad-spectrum antibiotics with piperacillin/tazobactam. Blood cultures were drawn and pending upon admission eventually growing *Staphylococcus Epidermidis* and *Staphylococcus Hominis*. The patient did not require vasopressor medications during her ED course. She was transferred to the medical intensive care unit for further management of severe sepsis with bacteremia, mediastinitis, and pleural effusions.

Following admission, cardiovascular-thoracic surgery consult deemed the patient a poor candidate for mediastinal debridement given her profound malnutrition and she was treated medically with an extended course of vancomycin. The PICC line was removed and initial left-sided pleurocentesis was described as a yellow, turbid pleural fluid without odor, similar in appearance to TPN or tube feeds. The mechanism by which TPN extravasation was greater on the left hemithorax than right remains unclear. Our patient required serial pleurocenteses during the course of a complicated 18-day hospitalization, and she was ultimately discharged to a skilled nursing facility with gastrostomy tube placement scheduled 12 days later. At the time of writing this report, the patient continued to experience extreme weight loss and complications from her prior gastric bypasses.

## DISCUSSION

Given the breadth of applications and safety profile, PICC lines are nearly ubiquitous in patients requiring long-term vascular access. Our knowledge of PICC line complications, however, remains incomplete, namely, the frequencies, risk factors, and mortality associated with tip migration and vessel perforation. A search of the literature showed this to be a rarely reported phenomenon,^5^,^6^ and to date there are limited studies detailing the prevalence of migrations or perforations in patients with PICC lines.^7^

Commonly reported PICC line complications include blood stream infections, occlusion, and thrombosis with tip migration being among the least reported.^8^ Catheter migration has been reported on average 43 days after placement^6^ with a caudal migration of 2.0 centimeters with abduction and adduction of the arm.^9^ Possible mechanisms for tip migration include coughing, vomiting, extreme physical activity, high-pressure infusions, and high-frequency ventilation.^10^ One case report proposed altered blood flow from repeated rapid changes in negative intrathoracic pressures and central venous pressures as a mechanism for tip migration in a patient receiving chemotherapy. The patient experienced vigorous coughing and vomiting spells likely resulting in forceful diaphragmatic contraction, alternating blood flow and subsequent tip migration from the superior vena cava to the right internal jugular vein.^11^ Other studies mention vessel perforation as a risk with placement of PICCs in the large vessels of the upper arm, asserting such complications are possibly iatrogenic, and caused during initial insertion or erosion after long-term use.

Proposed risk factors of PICC migration and perforation include the PICC line material, orientation, and insertion arm. Fernando et al note softer materials such as silicone or polyurethane likely allow for greater intravascular movement and possible migration but have lower rates of perforations.^14^ In contrast, less- compliant polyethylene catheters carry a higher risk of perforation. Malorientation of the migrated PICC line within the vessel is also considered a major risk factor for perforation. PICCs oriented obliquely or perpendicularly following migration are more likely to abut the vessel wall with changes in body positions and over time result in venous perforation.

Some researchers suggest that initial site of insertion may have predilections to vessel-specific migrations. For example, left-sided PICC line placement is an important risk factor for azygous migration,^12^ with an associated 19% complication rate for azygous perforation and resulting mediastinal and pleural effusions of transfused products.^7^ Other reported PICC-line vessel migrations include brachiocephalic, subclavian, and internal jugular veins (IJV), but no associations with initial-vessel insertions have been made.

Complications of PICC line migrations with perforations are not only mechanical but also physiologic in nature and carry the potential for high morbidity and mortality. A case report^13^ of an 80-year-old female experiencing immediate neck pain after vancomycin infusion through her left-sided PICC line led to a large fluid collection with rightward tracheal deviation, as well as a perforated left IJV. Despite confirmed optimal placement 11 days prior, the PICC line migrated and required removal. She experienced an uncomplicated hospital course thereafter.

Another case describes a fatal cardiac arrest after potassium-enriched solution was infused into the pericardial space via a perforated right-sided PICC, creating a rapidly progressive cardiac tamponade.^8^ A pericardial drain was placed, which reversed the patient’s hypotension; however, the authors hypothesize the transfused potassium diffused into the pericardium creating a local hyperkalemic state and terminal ventricular fibrillation.^6^ This case is particularly important in that it exemplifies a fatal physiologic response to appropriate transfusions in an inappropriate anatomical location. In our case the patient suffered from similar pathology with TPN inadvertently delivered into the mediastinum and pleural space, creating a nidus for bacterial proliferation and severe sepsis.

## CONCLUSION

As illustrated by previous case reports, there are a myriad of proposed mechanisms for and consequences of PICC migration and perforations that harbor lethal potential. Most reported complications were noted to have occurred in-patient, and of iatrogenic causes. Our case report, however, details a unique clinical scenario of a ground-level fall causing a traumatic PICC line migration and perforation of the right innominate vein resulting in mediastinitis. We present this case as a life-threatening PICC line complication that requires particular attention given its high associated mortality, but more importantly the location where the injury occurred. In this case, the patient was reported to have fallen at home with her evaluation initiated in the ED. Thus, emergency physicians need to be aware for the potential of such traumatic PICC line complications to facilitate prompt recognition and treatment when encountering patients with chronic indwelling vascular access.
